# Enhanced Adipose Mesenchymal Stem Cells Proliferation by Carboxymethyl-Chitosan Functionalized Polycaprolactone Nanofiber

**DOI:** 10.29252/ibj.24.4.236

**Published:** 2020-02-12

**Authors:** Atena Shapourzadeh, Seyed Mohammad Atyabi, Shiva Irani, Hadi Bakhshi

**Affiliations:** 1 Department of Biochemistry, Faculty of Basic Science, Islamic Azad University Damghan Branch, Damghan, Iran;; 2 Department of Pilot Nanobiotechnology, Pasteur Institute of Iran, Tehran 13164, Iran;; 3 Science and Research Branch, Islamic Azad University, Tehran, Iran;; 4 Universitatsstraße Bayreuth, Germany-Universitatsstraße Bayreuth, Germany

**Keywords:** Carboxymethyl chitosan, Mesenchymal stem cells, Tissue engineering

## Abstract

**Background:**

Through combining two synthetic and natural polymers, scaffolds can be developed for tissue engineering and regenerative medicine purposes.

**Methods:**

In this work, CMC (20%) was grafted to PCL nanofibers using the cold atmospheric plasma of helium. The PCL scaffolds were exposed to CAP, and functional groups were developed on the PCL surface.

**Results:**

The results of FTIR confirmed CMC (20%) graft on PCL scaffold. The MTT assay showed a significant enhancement (*p* < 0.05) in the cell affinity and proliferation of ADSCs to CMC20%-graft-PCL scaffolds. After 14 days, bone differentiation was affirmed through alizarin red and calcium depositions.

**Conclusion:**

Based on the results, the CMC20%-graft-PCL can support the proliferation of ADSCs and induce the differentiation into bone with longer culture time.

## INTRODUCTION

One of the most exciting goals in tissue engineering is regeneration and repair of bone injuries and damages^[^^1^^]^. Three constituents, including biomaterial scaffolds, stem cells, and planting technology *in vivo*, have a significant role in tissue engineering. Scaffolds can support cell proliferation and diffeentiation^[^^1^^]^. PCL is an aliphatic and a biocompatible polyester, and its main disadvantages are hydrophobicity and the slow rate of biodegradability. Hydrophobicity of PCL leads to the low cell loading at the initial stage of the cell culture, resulting in poor cell attachment and proliferation^[^^2^^,^^3^^]^.

The combination of biopolymers can improve the quality of a new scaffold. Various chemical or physical methods are availavle to link two different polymers^[^^4^^]^. Natural polymers, like collagen^[^^5^^]^, elastin^[^^6^^,^^7^^]^, gelatin^[^^8^^]^, and chitosan^[^^9^^-^^11^^]^, are usually grafted on PCL. Combined scaffolds have also been used to produce blood vessels^[^^9^^]^, nerve^[^^12^^]^, bone^[^^13^^]^, and skin repair agents^[^^14^^,^^15^^]^. 

As a natural polymer and an amino polysaccharide, chitosan is formed by β-(1,4)-2-acetamido-2-deoxy-D-glucose binary linear units. CMC is a hydrophilic modification of chitosan produced by carboxy-methylation. Biocompatibilities, biodegradability, as well as antifungal, antibacterial, and anticancer activities are the excellent properties of CMC^[^^16^^]^. 

One technique for the modification of physical surface is Cold Atmospheric Plasma, which is employed to create reactive functional groups on scaffolds with no change on the useful properties^[^^17^^]^. Ions, atoms, and free radicals are plasma-forming elements produced from gases like, oxygen^[^^6^^]^, nitrogen^[^^7^^]^, argon^[^^8^^]^, and helium^[^^9^^-^^11^^]^. Recently, many researchers have used various CAP devices for surface modifications^[^^18^^]^. Atyabi *et al.*^[^^19^^]^ have shown that fibroblast cells demonstrate a considerable growth and proliferation in the PCL modified by CAP. Meghdadi *et al.*^[^^20^^]^ have introduced a primary carboxyl group on the PCL surface by CAP treatment so that gelatin could graft on PCL through covalent attachment. 

In this study, helium gas was utilized to produce jet plasma needed for the grafting process. Through CAP treatment, carboxyl and hydroxyl groups were introduced on the PCL nanofiber surface to make grafting of CMC 20% on PCL possible. Moreover, the physical and morphological properties of the produced scaffolds were determined. In the following, adhesion and proliferation of ADSCs on CMC 20%-graft-PCL were assessed.

## MATERIALS AND METHODS

**Materials**

Chitosan extracted from crab shell with a degree of deacetylation of >90% was procured from Bio Basic (Canada). The PCL with the average molecular weight of 80,000 g moL^-1^ was purchased from Sigma-Aldrich (Germany). ADSCs were obtained from the Stem Cell Technology Research Center, Iran^[^^21^^]^ and DMEM, FBS, and Trypsin/EDTA solution (0.25%) from Gibco (Germany).

**Preparation of PCL by electrospinning**

To fabricate scaffold, PCL solution (12.5 wt%) was prepared in a mixture of chloroform/dimethyl-formamide (2/3 v/v) at room temperature. After that, electrospinning (CO881007NYI machine, Asia Nanostructure, Iran) process with a flow rate range of 0.1-0.7 mL/h, a voltage of 18-30 kV, and a needle-to-collector distance of 16-20 cm was used to produce scaffold. All scaffolds were collected on aluminum sheets rolled over the collector.

**Preparation of CMC**

Following Sharifi *et al.*^[^^21^^]^, chitosan (1 g) was first dissolved in acetic acid/deionized water mixture (1/9 v/v) with continuous stirring at room temperature overnight. Sodium hydroxide was added to precipitate out the chitosan. The precipitated chitosan was isolated and washed with deionized water and isopropanol. The purified chitosan was dissolved in isopropanol and sodium hydroxide and stirred for five hours. Afterwards, a mixture of monochloroacetic acid and isopropanol was added dropwise and stirred at room temperature for eight hours. Finally, the precipitate was filtered, washed with ethanol and dried in a vacuum oven. 

**CAP treatment**

The CAP was generated at 15 MHz at a radio frequency of 10 kV with 10 W output power using the round, as an external electrode, and helium gas flow of 5 L/min. The distance between the plasma jet nozzle and the sample surface was 2 cm. To introduce polar hydrophilic groups such as hydroxyl and carboxyl on PCL, the samples were treated by CAP in 60^th^ and 90^th^ seconds. It is essential to determine the most appropriate time period to maintain the morphology of the scaffold. 

**Contact angle**

To analyze the hydrophilicity surface of PCL, water contact angle was measured by one water droplet (5 μL) on the surface of the nanofiber for 1 minute (DataPhysics, Germany). 

**Preparation CMC-graft-PCL**

To fabricate CMC-graft-PCL, PCL was exposed to CAP for an optimum period of time, and the treated PCL was immobilized on glutaraldehyde. Afterwards, CMC was dissolved in DDW for 3 h. Then 400 μL of the CMC-DDW solution was added onto the treated PCL in a Petri dish. After the plasma coupling reaction, the CMC connected to PCL was immersed into DDW for 24 h and then dried in vacuum. Finally, the scaffolds of CMC20%-graft- PCL were prepared. 

**Characterization of scaffold**

***Scanning electron spectroscopy***

Scanning electron spectroscopy^[3]^ was applied to investigate the morphology and structure of PCL nanofibers as well as determine the suitable CAP exposure time. The sample was sputter-coated by a gold layer and then examined by a VEGA/TESCAN (SEM, VEGA, TESCAN, Czech Republic) scanning electron spectroscopy at an accelerating voltage of 2 kV.

***FTIR analyses***

To investigate the chemical modification of PCL, PCL + CAP and CMC20%-graft-PCL were characterized using Equinox 55 ATR-FTIR spectrometer (Bruker, Germany). The spectra were recorded from 400 to 4000 cm^-1^ with a 4-cm^-1^ resolution.

***Cell culture ***

The ADSCs were maintained in DEME (Gibco) supplemented with 10% FBS (Gibco) at 37 °C and 5% CO_2_and passaged every 3-4 days depending on the cell proliferation and confluence. The cell viability was evaluated qualitatively by an inverted microscope (Bell, INV-100FL, Japan). For cell passages, the cells were washed with PBS solution and treated with Trypsin (0.25%) and EDTA (0.1%) at 37 °C for 2 min. The detached cells were collected by centrifugation and resuspended in DMEM + 10% FBS. 

***Cell viability assay***

Cell viability of ADSCs on CMC20%-graft-PCL was analyzed using MTT (Sigma-Aldrich) assay. After three passages, an initial density of 1 × 10^4^ cells/cm^2^ of ADSCs was seeded on 96-well tissue culture plates. The CMC20%-graft-PCL was cut into 0.5 × 0.5 cm^2^ and sterilized under a UV lamp (both sides) for 40 min. Sterilized scaffolds were placed on a 96-well plate in triplicate on which ADSCs were seeded (10^4^ cells/well) and incubated for 24, 48, and 72 h. There was also a one-well plate without scaffolds as a control. After incubation, the culture medium of each sample was replaced with 200 μL of MTT solution (5 mg/mL) and incubated for 4 h. Then the MTT solution was removed and replaced by 60 μL of DMSO for 5 minutes at room temperature, and the OD of the wells was determined by a spectrophotometer at 570 nm. The results were analyzed using analysis of variance (ANOVA) in SPSS software (*p* < 0.05). 

***Alizarin red staining assay***

For alizarin red staining, the attached cells were washed with cold PBS and fixed in cold 4% paraformaldehyde solution at 4°C for 20 min. After being washed with PBS, the fixed cells were stained by 2% alizarin red solution for 5-10 min. Finally, the stained cells were washed with PBS and examined under an inverted light microscope.

***Calcium content assay***

The number of calcium minerals deposited by ADSCs on scaffolds after 14 days of incubation was counted using the o-Cresolphthalein method^[^^22^^]^. Calcium extraction was performed by homogenization of the scaffolds in 0.6 N of HCL, followed by shaking at 4 °C for 4 h. OD was measured using a calcium content kit (Pars Azmun, Iran) at 550 nm after adding the reagent to calcium solutions. Calcium content was obtained according to the standard curve of OD versus a serial dilution of calcium concentrations. 

**Statistical analysis**

All the experiments were conducted at least three times (n = 3). The data were evaluated and compared using one-way analysis of variance (ANOVA; *p* < 0.05).

## RESULTS

**Characterization of PCL-treated CAP**

**Fig. 1:**
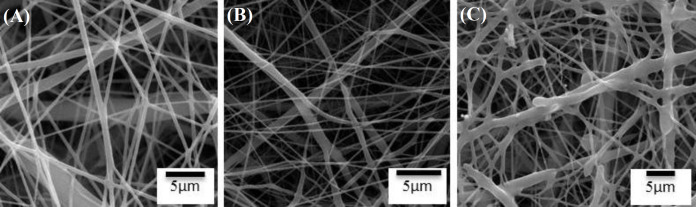
SEM images of (A) untreated, (B) CAP-treated (at 60 s), and (C) CAP-treated (with 90 s) PCL scaffolds

The SEM imaging of electrospun PCL nanofibers was used to examine the morphology of the treated PCL (Fig. 1). As shown in the Figure, PCL scaffold kept its nanofibrous morphology after 60 s exposure to CAP. Moreover, the fibers were bead-free, with randomly oriented smooth surface, morphology. However, with increased exposure time, the nanofibers were melted. As a result, the optimum time of CAP treatment was equal to 60 seconds.

**Water contact angle**

Hydrophilicity and surface wettability of the CL have been shown to affect cell adhesion^[^^19^^]^. The contact angle of untreated PCL nanofiber was 120 ± 5˚ and higher than that of PCL-treated CAP (18 ±5˚), indicating that the treated CAP can notably improve the hydrophilicity of the PCL surface.

**Fabrication of CMC-graft-PCL**

***FTIR analyses***

**Fig. 2:**
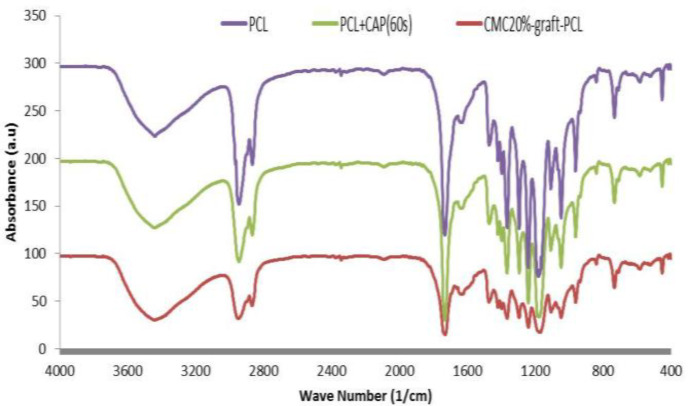
FTIR spectra of PCL, CAP-treated PCL scaffold with 60 s, and CMC20%-graft-PCL

To ensure that CMC is grafted on the PCL, ATR-FTIR technique was implemented (Fig. 2). The CMC20%-graft-PCL was carried out through the mentioned method and compared with the CAP-treated and -untreated samples; three distinct peaks were observed in FTIR spectra. The peaks at 3435 cm^-1^ and 1604 cm^-1^ in a CMC20%-graft-PCL sample can be attributed to the stretched hydroxyl (−OH) and amino group (N–H)^[^^21^^,^^23^^]^. The untreated PCL spectrum includes the stretching vibration of carbonyl bond (C=O) at about 1730 cm^−1^, C–H bonds at 2948 and 2867 cm^-1^, and ester groups at 1181 cm^-1[^^21^^,^^24^^,^^25^^]^. Apperance of a sharp peak near 1727 cm^−1^ according to the carbonyl stretching (C=O) was considered as CAP-treated PCL spectrum. Based on the results of FTIR spectra, the CMC20% graft on the CAP-treated PCL surface was confirmed.

**Cell adhesion and viability**

**Fig. 3:**
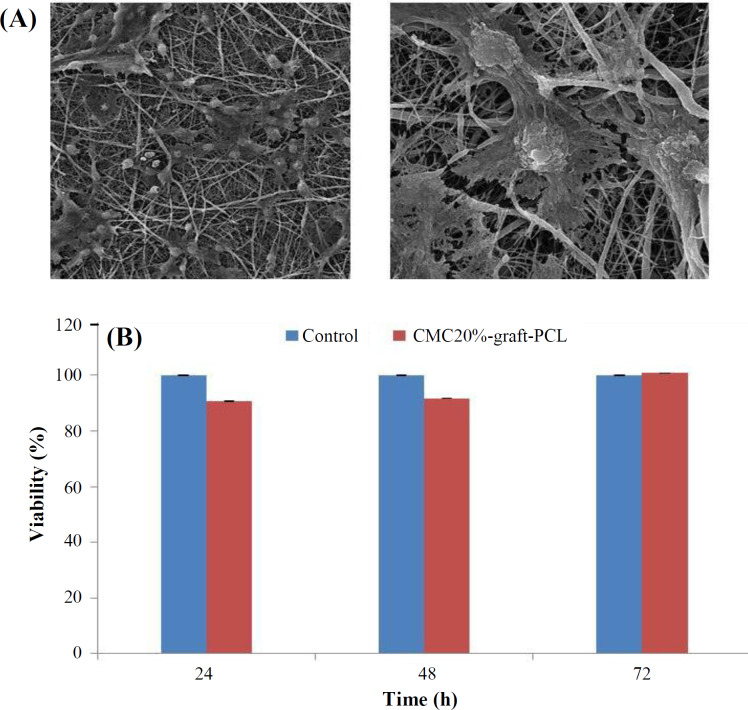
(A) SEM images of ADSCs attached to the CMC20%-graft-PCL scaffolds. (B) Viability of ADSCs on the scaffolds after 24-72 hours of incubation based on MTT assay. Plate wells without scaffold were used as the control. The data showed no significant differences (*p* ≤ 0.05)

The biological response of the CMC20%-graft-PCL scaffolds was evaluated through cytotoxicity and cell attachment. *In vitro *cytotoxicity of the scaffolds was determined by placing them in direct contact with ADSCs for 72 hours. The shape of cells, spreading, and orientation on the CMC20%-graft-PCL scaffolds are illustrated in the SEM images (Fig. 3). Given the micrographs of the SEM, CMC20%-graft-PCL scaffolds are featured with higher cell attachment and density. Therefore, CMC20%-graft-PCL nanofibrous scaffolds demonstrate a higher cell integration (Fig. 3A). The usefulness of scaffolds for tissue engineering application was studied by culturing cells on their surfaces. For this purpose, ADSCs were cultured on the CMC20%-graft-PCL for 24, 48, and 72 hours, and their viability was determined using MTT assay (Fig. 3B). After three days, the cell growth and proliferation were close to the control sample (without scaffolds; *p* > 0.05). Therefore, the CMC20%-graft-PCL was a suitable scaffold for osteoblast cell attachment and proliferation.

***Alizarin red staining assay***

**Fig. 4:**
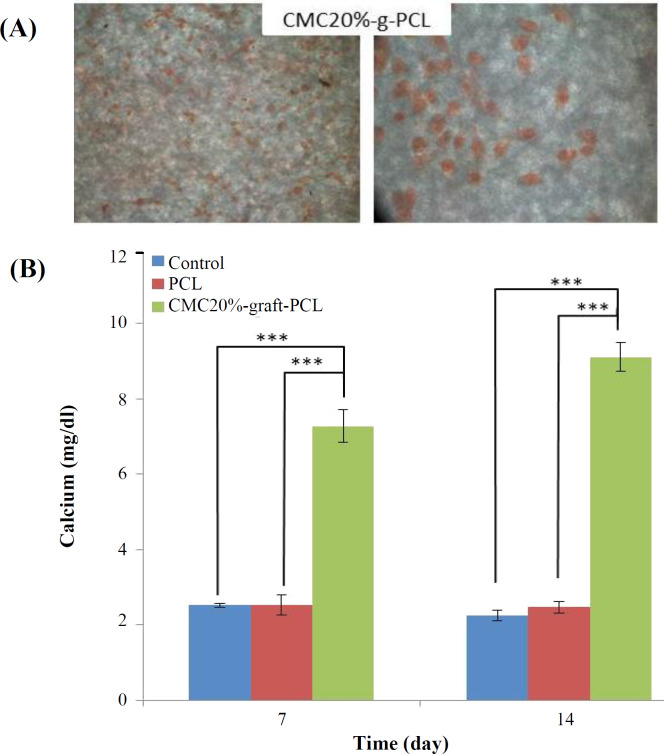
(A) Alizarin red staining of cells on the CMC20%-graft-PCL scaffold (5 × 5 mm^2^) after seven days of incubation. (B) The calcium content (n = 3) of the CMC20%-graft-PCL scaffold (5 × 5 mm^2^) and PCL after 7 and 14 days of incubation. The initial content of seeded ADSCs was 10^4^ cells. The tissue culture plate was used as the control. ^***^p ≤ 0.001

Alizarin red staining was performed to qualitatively evaluate the formation of the mineralized matrix on the scaffolds. The results indicated the absorption of alizarin red dye on CMC20%-graft-PCL scaffolds, which was due to the calcium deposed by osteodifferentiated cells (Fig. 4A). 

***Calcium content assay***

The volume of calcium produced by the differentiated cells was measured using calcium content assay. As shown by the results, there was an increase in the volume of calcium deposited on CMC20%-graft-PCL scaffolds in 14 days (*p* < 0.05; Fig. 4B).

## DISCUSSION

According to studies, a weakness of PCL scaffold, which decreases the adhesion of cells on its surface, is the intrinsic hydrophobic property, and, consequently, lowering the cell-scaffold interaction^[^^26^^]^. One of the exciting solutions to this problem is to combine this scaffold with another natural polymer Here, CAP treatment on the PCL surface was used to graft CMC. Through CAP, rebuilding the microstructures and modifying the surface properties of the scaffolds became possible^[^^17^^,^^20^^]^. 

Based on the SEM image (Fig. 1) of CAP-treated PCL, nanofibrous scaffolds showed that the optimum treatment time was 60 s. During this time perid, no nanofibers morphology alteration or damage to the structure of the PCL was observed. The CAP added oxygen-containing groups like carboxyl, which resulted in a higher hydrophilicity and surface enhancement for grafting. Consequently, wettability and coloniality by cells were improved^[^^20^^,^^27^^] ^. Bak *et al.*^[^^27^^]^ have demonstrated that the PCL is treated by O_2_ or N_2_ plasma, therby leading to hydrophilicity. The plasma promotes the formation of free radicals that can act as interlock points for active species such as polar groups. Many studies have used various plasma treatment techniques with different exposure times^[^^18^^,^^28^^]^. Meghdadi *et al.*^[^^20^^] ^have suggested that PCL nanofibers are melted by exposing to helium plasma for more than 3 min (5 and 7 min). Comparing with our results, their exposure time was more than 1 min longer.

Regarding the water contact angle, there was an improvement in the surface hydrophilicity of scaffolds as a result of using helium plasma for surface treatment. In addition, a contact angle of untreated- PCL scaffold was 18 ± 5°. The CAP influenced the chemical group on PCL and induced changes in surface topography, so that it had a positive effect on the wettability^[^^29^^,^^30^^]^. However, Trizio *et al.*^[^^28^^]^ did not report the absorption of water in helium CAP-treated PCL. This behavior might be due to the time of exposure and type of plasma device. We achieved the hydrophilicity of PCL by using helium CAP for 60-s treatment. The FTIR results of the CMC-grafted PCL (Fig. 2) confirmed the formation of functional groups on the surface of PCL nanofibers, which expresses polymeric chains linkage of PCL with CMC. The combination of CMC and PCL led to an improvement in the cellular attachment and proliferation. Sharifi *et al.*^[^^21^^]^ fabricated the PCL/CMC scaffolds using blend electrospinning method for bone tissue engineering applications. To this end, human osteoblast cells (MG63) were cultured on the scaffolds. Their results showed that CMC promoted proliferation, and it was an excellent material for bone tissue engineering. Alemi *et al.*^[^^31^^]^ made PCL/CMC10% scaffold by blend electrospinning method. They mixed PCL solution with 10% solubilized CMC. Their results revealed that the PCL/CMC scaffolds supported and induced the differentiation of mesenchymal stem cells to cartilage like cells. It is noteworthy that CMC20%-graft-PCL was fabricated through a physical technique rather than blend electrospinning methods. 

To assess the viability and growth rate of ADSCs on the CMC20%-graft-PCL, the cell growth was evaluated by MTT assay for 72 h. As illustrated in Figure 3B, the viability of ADSCs on CMC20%-graft-PCL increased during all culture times, compared to the control group (*p* > 0.05). Although insignificant, these differences reflect the extreme tendency of ADSCs to the CMC20%-graft-PCL scaffold. Since cell adhesion and proliferation are essential for differentiation, the CMC20%-graft-PCL scaffold can contribute to bone tissue engineering. The use of scaffolding synthesis materials plays a central role in the success of bone tissue engineering^[^^31^^]^. A recent study has provided evidence that gelatin/CMC scaffoldsand laponite-incorporated scaffolds (10%) induce a higher degree of osteogenic differentiation of recombinant bone marrow stromal cells^[^^32^^]^ compared to the GC scaffold and GC-Lap5% scaffold^[^^33^^]^. Alizarin red and calcium content tests indicated that CMC20% incorporated in scaffolds had an osteoinductive effect on the differentiation of ADSCs to osteoblasts, where no external osteogenic differential agent was used. Moreover, low calcium content was detected for PCL scaffold, showing no differentiation of ADSCs to osteoblasts.

Overall, our findings showed that the CMC20%-graft-PCL has ability to provide cellular adhesion and proliferation and induce the differentiation of ADSCs to bone upon longer culture time. Moreover, the incorporation of CMC to PCL fibers could provid a more promising scaffold for bone tissue engineering applications.

## CONFLICT OF INTEREST.

None declared.
